# Anti-Aging Efficacy of Fructosazine and Deoxyfructosazine: A Comprehensive In Vitro and In Vivo Analysis

**DOI:** 10.3390/molecules30112263

**Published:** 2025-05-22

**Authors:** Ao Yang, Chunyan Ma, Qiling Song, Wenhui Li, Shixuan Lv, Xiuhan Guo, Shisheng Wang, Zhigang Gao, Shuai Wang, Qingwei Meng, Yueqing Li

**Affiliations:** 1 State Key Laboratory of Fine Chemicals, Department of Pharmaceutical Engineering, School of Chemical Engineering, Dalian University of Technology, 2 Linggong Road, Dalian 116024, China; yangao991220@126.com (A.Y.); mcy1226@126.com (C.M.); lsql2000@dlut.edu.cn (Q.S.); azaztata@163.com (W.L.); 15731693403@163.com (S.L.); kakumicin@dlut.edu.cn (X.G.); wangss@dlut.edu.cn (S.W.); gzg1980@dlut.edu.cn (Z.G.); mengqw@dlut.edu.cn (Q.M.); 2 Ningbo Institute of Dalian University of Technology, No. 26 Yucai Road, Jiangbei District, Ningbo 315016, China; 642805803@163.com; 3 Liaoning Binhai Laboratory, Dalian 116023, China

**Keywords:** skin, anti-aging, fructosazine, deoxyfructosazine, skincare

## Abstract

As the proportion of the elderly population increases, there is an urgent need for anti-aging technologies. Since the skin is the most visibly aging organ in the human body, it is crucial to develop active ingredients to slow down skin aging. Currently, identified anti-aging active substances include antioxidants, retinoids, peptides, growth factors, and compounds derived from biofermentation. However, they have limitations such as poor stability, low transdermal permeability, skin irritation, high effective concentrations, slow onset of efficacy, single-action mechanisms, and high production costs. These limitations highlight the necessity of developing new anti-aging technologies that are multifunctional and cause low irritation. This study aimed to investigate the anti-aging effects and mechanisms of fructosazine (FZ) and deoxyfructosazine (DOF) on the skin as well as their potential applications in skincare. The methods included ELISA tests to assess the viability of human dermal fibroblast (NHDF) cells and related factors, and monitoring in Sprague-Dawley (SD) rats. The results showed that FZ promoted cell viability. Both FZ and DOF enhanced the secretion of type I collagen (Col I) and hyaluronic acid (HA), inhibited matrix metalloproteinase-1 (MMP-1), boosted catalase (CAT), and reduced malondialdehyde (MDA), reactive oxygen species (ROS), and β-galactosidase. They also nourished the epidermis and increased fiber content. In conclusion, FZ and DOF can stimulate the production of anti-aging substances, exhibit antioxidant activity, and have potential in skincare.

## 1. Introduction

Aging is a complex and inescapable biological process, which is manifested by the gradual decline of organ functions over time and is influenced by numerous biochemical reactions and environmental factors [1]. For example, the oxidation-reduction reaction is one of the important biochemical reactions in the body. During the aging process, the balance of oxidation and reduction in cells is disrupted. Excessive production of reactive oxygen species (ROS) can damage cellular components such as DNA, proteins, and lipids. Another example is the glycation reaction. Glucose in the body can react with proteins non-enzymatically, forming advanced glycation end products (AGEs). The accumulation of AGEs can lead to the cross-linking of proteins, thereby affecting the normal function of tissues and organs. These biochemical reactions interact with each other and jointly promote the progress of aging. The skin, being the largest and most complex organ in our body and accounting for approximately one-sixth of body weight, plays a crucial role in our health and well-being [2]. It functions as a protective barrier, regulates body temperature, retains moisture, enables sensory perception, and supports immune surveillance. Skin aging, the most visible indication of the body’s aging process, is characterized by a variety of symptoms such as skin laxity, wrinkling, dryness, loss of elasticity, pigmentation, and thinning [3].

Skin aging is a natural process determined by genetic factors and influenced by various environmental factors, and includes endogenous aging (natural aging) and exogenous aging. The endogenous aging process is affected by both genetic and endocrine factors. The number of dermal fibroblasts decreases, and the ability to synthesize collagen and elastin (especially type I and type III collagen) in the extracellular matrix (ECM) declines, resulting in thinning of the epidermis and dermis, skin atrophy, fine wrinkles, and dryness, etc. [4,5]. Exogenous aging is induced by external environmental factors, such as ultraviolet radiation, air pollutants, smoking, temperature, and diet, which lead to a series of skin aging problems, including deeper wrinkles, skin laxity, the formation of pigment spots, and telangiectasia [6]. When the skin is exposed to ultraviolet radiation for extended periods, keratinocyte activity decreases, epidermal renewal slows down, the epidermal barrier function weakens, the amount of amorphous elastic fibers increases while collagen is destroyed and disorganized, and the number of Langerhans cells decreases. These lead to a decline in skin immune function and, compared with endogenous aging, result in irregular pigmentation, telangiectasia, rough skin, and deep wrinkles [7,8].

At present, classic theories on the mechanism of skin aging include the theory of free radicals and oxidative stress, the theory of inflammatory aging, the theory of skin photoaging, and the theory of nonenzymatic glycosyl chemistry, and others [9]. Since the framework of skin aging mechanisms has been initially formed, a variety of skin anti-aging strategies have emerged accordingly. (1) Antioxidants: Antioxidants (such as vitamin C, vitamin E, coenzyme Q10, etc.) protect skin cells from oxidative damage and reduce oxidative stress reactions by directly participating in the neutralization reaction of free radicals or stabilizing free radicals as electron donors [10]. Astaxanthin has antioxidant and anti-inflammatory effects and can reduce skin photoaging [11]. (2) AGE inhibitors: 4,5-Dimethyl-3-phenylthiazolium chloride (ALT-711), an AGE cross-link-breaking agent, can break existing AGE-induced cross-links in collagen, may restore tissue elasticity, and has been studied for its potential to improve vascular function in aging and hypertension [12]. Natural plant extracts (such as quercetin, curcumin, green tea polyphenols, resveratrol, etc.) show good anti-glycosylation effects and have the role of delaying skin aging [13]. (3) Natural anti-inflammatory components: Natural plant anti-inflammatory components (such as curcumin, triptolide, ursolic acid, etc.) are increasingly widely used in skincare products. These components have significant anti-inflammatory effects, relieve skin allergic reactions, and delay the skin-aging process caused by inflammatory factors [14,15]. (4) Components for improving the ECM microenvironment of the dermis: Retinol can significantly improve the microenvironment of the dermal ECM, stimulate skin cells to produce ECM (such as type I collagen, fibronectin and elastin), promote the formation of dermal blood vessels, and stimulate TGF-β/CTGF, which are the main regulatory factors for maintaining ECM homeostasis [16]. Collagen-derived tripeptide (Gly-Pro-Hyp) can prevent the reduction of ECM-related genes COL1A, elastin, and fibronectin [17]. C-Xyloside causes an increase in glycosaminoglycan synthesis in human dermal fibroblasts in culture, and this increase may lead to the maintenance of matrix integrity, thus contributing to the improvement of skin firmness [18]. With the advancement of scientific research and technological and methodological innovations in recent years, new skin anti-aging strategies will continue to emerge in the future.

**Figure 1:**
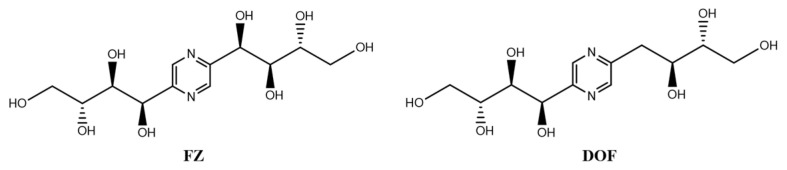
The structures of FZ and DOF.

Fructosazine (FZ) and deoxyfructosazine (DOF) (Figure 1), belonging to the class of polyhydroxyalkylpyrazines (PHAPs), have been identified in roasted food products and extracts of traditional Chinese medicine [19,20,21,22]. Research into the biological activities of PHAPs is currently limited, with only a handful of studies reporting on their properties. Some investigators have observed that both FZ and DOF exhibit antibacterial activity [22,23] and could induce DNA strand breaks [24]. Moreover, these compounds are hypothesized to have immunomodulatory potential, although specific mechanisms remain to be elucidated. In addition to their antimicrobial effects, FZ has been proposed as a therapeutic agent for the management of osteoarthritis and rheumatoid arthritis. DOF has been shown to suppress the production of interleukin-2 (IL-2) in T cells, suggesting an application in modulating immune responses [25]. Furthermore, DOF has demonstrated efficacy in the treatment of diabetes, indicating its multifaceted therapeutic potential [26].

In our anti-aging research, FZ was unexpectedly synthesized and found to exhibit a certain delaying effect on the natural aging of human embryonic lung diploid cells (2BS cells) [27]. On one hand, the multi-hydroxyl characteristics and hygroscopicity of FZ resemble the multi-hydroxyl structure of the star cosmetic raw material, Pro-Xylane. On the other hand, 2BS cells are also a type of fibroblast. These two aspects sparked our interest in exploring its use as a cosmetic raw material based on NHDF cells (human skin fibroblast cells). Considering that DOF is a compound with a very similar structure to FZ and relatively easier to synthesize, we included DOF in our research.

This study aims to explore the anti-aging effects of FZ and DOF on the skin and their underlying mechanisms. It also attempts to fill the research gap in the anti-aging properties of FZ and DOF and provide a scientific basis for their potential application in anti—aging skincare products. Pro-xylane, an anti-wrinkle ingredient widely used in the cosmetics market, was selected as a reference product to compare the effects of FZ and DOF. The study found that the anti-aging effects of FZ and DOF on the skin are better than those of Pro-xylane. FZ and DOF have strong anti-aging properties for the skin, such as stimulating the vitality of dermal fibroblasts, promoting the secretion of collagen and hyaluronic acid, inhibiting the expression of matrix metalloproteinase-1, and reducing oxidative stress. Therefore, FZ and DOF exhibit a combined anti-aging effect through multiple mechanisms of action. They may have broad application prospects in the field of anti-aging cosmetics in the future.

## 2. Results

### 2.1. Effects of Compound Treatments on Cell Viability

**Figure 2:**
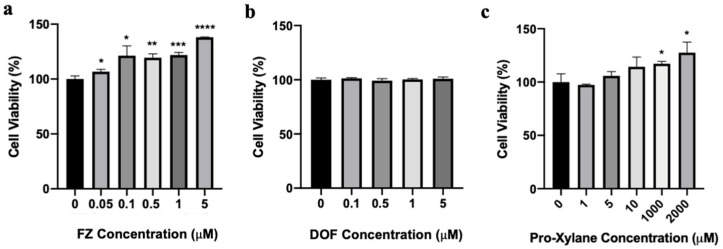
Impact of compounds on the viability of normal cells. (**a**) The effect of Fructosazine on cell viability. (**b**) The effect of Deoxyfructosazine on cell viability. (**c**) The effect of Pro-xylane on cell viability. n = 3 in each group. * *p* < 0.05; ** *p* < 0.01; *** *p* < 0.001; **** *p* < 0.0001.

A prominent hallmark of skin aging is thinning of the skin, often attributed to a decline in the number of skin cells [3,28]. We investigated the effects of drug treatment on cell viability. At concentrations of 0.1 μM to 5 μM, FZ significantly enhanced the viability of NHDF cells, increasing cell viability to 119.4% and 138.1%, respectively, with the most pronounced effect observed at 5 μM (Figure 2a). Within the same concentration range, DOF neither exerted toxicity nor enhanced cell viability on NHDF cells (Figure 2b). Pro-xylane was non-toxic to NHDF cells across the concentration range of 1 μM to 2 mM and displayed a slightly enhancing effect on cell viability at concentrations of 5 μM to 2 mM (Figure 2c).

### 2.2. Effects of Compound Treatments on the Secretion of Col I, HA, and MMP-1 in Normal Cells

**Figure 3:**
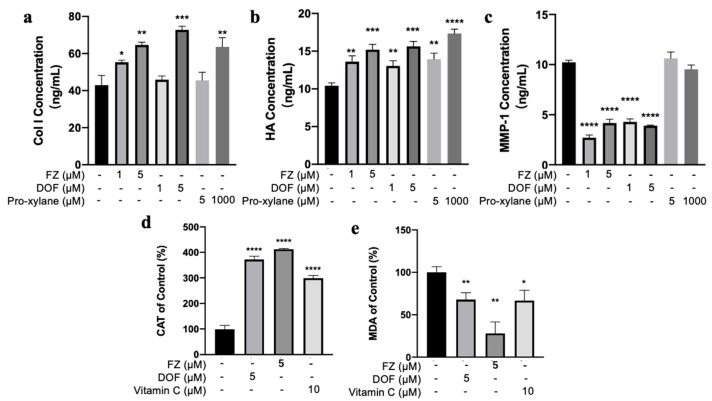
Effects of compounds on the levels of Col I (**a**), HA (**b**), MMP-1 (**c**), CAT activity (**d**), and MDA content (**e**) in normal cells. n = 3 in each group. * *p* < 0.05; ** *p* < 0.01; *** *p* < 0.001; **** *p* < 0.0001, when compared with the control group.

Collagens (Col) maintain skin elasticity and firmness. As people age, there is a significant loss of collagen in the body, which leads to symptoms such as skin laxity [28]. Hyaluronic acid (HA), a natural moisturizing factor, effectively hydrates the skin. When hyaluronic acid in the skin diminishes, the skin may become dry and undergo keratinization [29]. Matrix metalloproteinases (MMPs) are the primary proteases responsible for collagen fiber breakdown and are found at elevated levels in aged skin [30]. We examined the effects of drug treatment on the secretion of Col I, HA, and MMP-1 in cells under normal culture conditions. Compared with the control group, both FZ and DOF promoted the secretion of Col I and HA and suppressed the secretion of collagen-degrading MMP-1 in NHDF cells under normal culture conditions. Specifically, 1 μM and 5 μM FZ increased Col I secretion by 128.8% and 150.7%, respectively; increased HA secretion by 130.5% and 145.7%; and reduced MMP-1 levels to 26.4% and 40.8% of the normal levels. Similarly, at concentrations of 1 μM and 5 μM, DOF increased Col I secretion by 106.9% and 169.6%, respectively; increased HA secretion by 125.1% and 150.0%; and reduced MMP-1 levels to 38.2% and 41.9%. Meanwhile, at concentrations of 5 μM and 1 mM, Pro-xylane increased Col I secretion by 106% and 148.1%, respectively; increased HA secretion by 133.7% and 166.3%; but had a minor inhibitory effect on MMP-1 (Figure 3a–c). This test result indicates that FZ and DOF have an advantage in these aspects. At the same concentration, the activities of FZ and DOF are superior to those of Pro-xylane.

### 2.3. Effects of Compound Treatments on CAT Activity and MDA Content in Normal Cultured Cells

Catalase (CAT) is a naturally occurring and crucial antioxidant enzyme in the human body. It catalyzes the self-redox reaction of superoxide anion radicals, effectively scavenging free radicals and maintaining the dynamic equilibrium between free radicals and redox states in the body, thus preventing cellular damage [31]. Free radicals can trigger peroxidation reactions on lipids within the body, producing malondialdehyde (MDA). MDA is cytotoxic and can induce cross-linking and polymerization of biomacromolecules such as proteins and nucleic acids, interfering with their normal functions [32].

We assessed the effects of compounds on CAT activity and MDA levels in cells under normal culture conditions. The results revealed that 5 μM FZ elevated CAT activity by 412.2% and cut MDA content down to 28.2% in normally cultured NHDF cells. Likewise, 5 μM DOF augmented CAT activity to 372% of the baseline and reduced MDA content to 67.9%. For comparison, 10 μM vitamin C escalated CAT activity to 299% and diminished MDA content to 66.7% (Figure 3d,e). These findings indicate that the antioxidant efficacy of FZ and DOF significantly exceeds that of the positive control, vitamin C, in normal cultured cells.

### 2.4. Effects of Compound Treatments on Cell Viability of Oxidatively Stressed Cells

**Figure 4:**
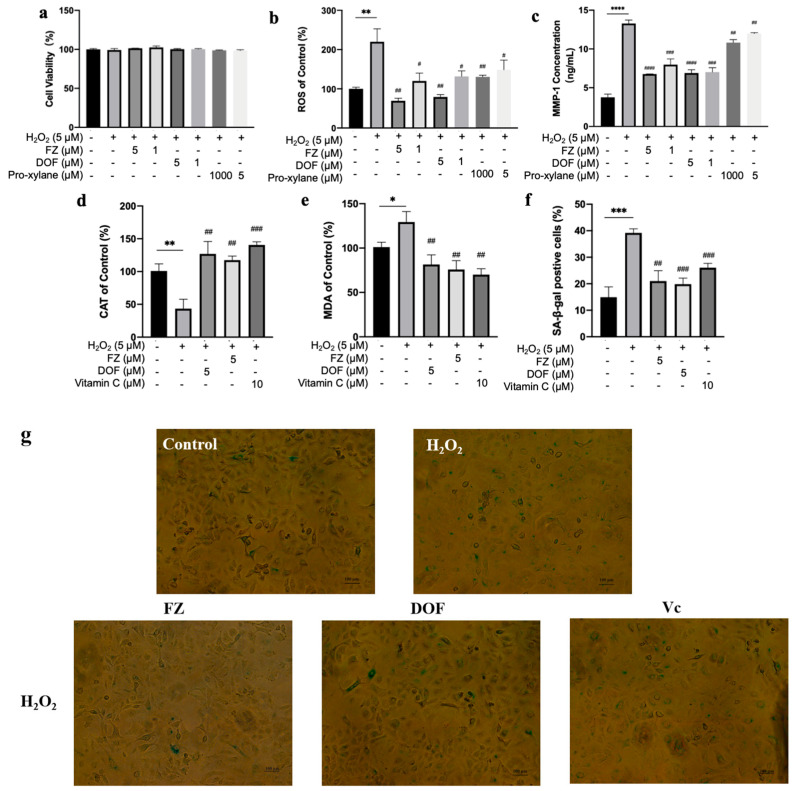
Effects of compounds on the viability (**a**), ROS levels (**b**), MMP-1 secretion (**c**), CAT activity (**d**), MDA content (**e**), SA-β-gal expression (**f**), and cell morphology (**g**) in oxidatively stressed cells. n = 3 in each group. * *p* < 0.05; ** *p* < 0.01; *** *p* < 0.001; **** *p* < 0.0001, when compared with the control group. ^#^ *p* < 0.05; ^##^ *p* < 0.01; ^###^ *p* < 0.001; ^####^ *p* < 0.0001, when compared with the H_2_O_2_ group.

The deleterious effects of reactive oxygen species (ROS) on cellular health are well documented. They can cause irreversible damage and even cell death [33]. In a relevant literature [34], treating NHDF cells with 5 μM H_2_O_2_ does not lead to severe cell damage and can produce a useful oxidative stress cell model. Previous studies (as shown in Figure 2a,b) have indicated that neither FZ nor DOF at concentrations of 1 μM and 5 μM exerts a toxic effect on NHDF cells. Based on these observations, we used 5 μM H_2_O_2_ as an inducer of oxidative stress to explore its impact on cell survival and simultaneously evaluate the potential protective effects of pretreating with different compounds. Notably, even after being exposed to 5 μM H_2_O_2_ for 24 h, the cells showed no signs of damage. Similarly, pretreating oxidatively stressed cells with 1 μM or 5 μM of either FZ or DOF, as well as 5 μM or 1 mM of Pro-Xylane, did not induce any additional toxicity (Figure 4a). Consequently, in subsequent experimental designs, we adopted concentrations of 5 μM H_2_O_2_, 1 μM and 5 μM of either FZ or DOF, and 5 μM and 1 mM of Pro-Xylane. This experimental strategy enables us to comprehensively explore the potential of these compounds to alleviate cell damage caused by oxidative stress.

### 2.5. Effects of Compound Treatments on ROS and MMP-1 Levels in Oxidatively Stressed Cells

Excessive reactive oxygen species (ROS) can escalate the expression of matrix metalloproteinases (MMPs), leading to collagen and elastin breakdown within the body, and subsequent skin wrinkling and sagging [35]. Our study probed into the impact of pretreatment with specific compounds on intracellular ROS levels and MMP-1 secretion in cells under oxidative stress.

While cells were subjected to oxidative stress, ROS levels soared to approximately 219.8% of normal levels. However, pretreatment with either FZ or DOF showed potent ROS scavenging capabilities. Precisely, 1 µM and 5 µM of FZ decreased ROS levels in oxidatively stressed cells to 31.5% and 54.5%, respectively. Likewise, 1 µM and 5 µM of DOF lowered ROS levels to 36% and 59.8%, respectively. Moreover, 5 µM and 1 mM of Pro-Xylane reduced ROS levels to 59.3% and 67.5%, respectively (Figure 4b).

Notably, the secretion of MMP-1 from oxidatively stressed cells spiked, reaching about 353.4% compared with normal levels. Pretreatment with 1 µM or 5 µM of FZ significantly decreased MMP-1 secretion from oxidatively stressed cells to 50.9% and 60.1%, respectively. Similarly, 1 µM or 5 µM of DOF reduced MMP-1 secretion to 52% and 52.9%, respectively. Treatment with 5 µM or 1 mM of Pro-Xylane led to a decline in MMP-1 secretion to 81.5% and 90.3%, respectively (Figure 4c).

These results emphasized that FZ and DOF effectively suppressed MMP-1 secretion from cells under oxidative stress, thereby reducing collagen breakdown and increasing collagen content within the body, achieving effects such as skin firming, wrinkle reduction, and anti-aging. From our findings, it was evident that, in terms of managing ROS levels and MMP-1 secretion, FZ and DOF outperformed Pro-Xylane, which is currently popular in the market. They showed potential as powerful agents against skin aging caused by oxidative stress, holding promise for improvements in skin health and appearance.

### 2.6. Effects of Compound Treatments on SA-β-gal Expression and Cell Morphology in Oxidatively Stressed Cells

The expression of senescence-associated β-galactosidase (SA-β-Gal) is upregulated in senescent skin cells [36]. We investigated the impact of compound treatments on SA-β-Gal expression and cellular morphology in oxidatively stressed cells. In comparison with the control, oxidatively stressed cells showed a significant increase in blue staining, reaching approximately 263.1% of the normal levels, and presented senescent characteristics such as loose arrangements and blurred cell edges. However, treatment with FZ, DOF, or vitamin C decreased the percentage of senescent cells within oxidatively stressed cells to 53.6%, 50.8%, and 66.6%, respectively, alleviating oxidative damage, maintaining cellular morphology, and delaying senescence (Figure 4f,g).

### 2.7. Effects of Compound Treatments on CAT Activity and MDA Content in Oxidatively Stressed Cells

CAT can swiftly scavenge harmful substances like H_2_O_2_ produced by cellular metabolism through binding with mitochondria and peroxisomes in cells, thereby decreasing the generation of free radicals and mitigating oxidative damage [31]. MDA is one of the cellular products resulting from lipid peroxidation, and its formation can exacerbate membrane injury [32]. By measuring MDA levels, we can indirectly gauge the extent of membrane damage and the organism’s stress-resistance capacity. We examined the effects of compound treatments on CAT activity and MDA levels in oxidatively stressed cells. Compared with the control group, oxidatively stressed cells showed a notable decrease in CAT activity, which was about 43% of normal CAT activity, while MDA levels rose to roughly 129.3% of normal levels. However, treatments with FZ or DOF alleviated oxidative damage, decreased lipid peroxidation, and enhanced CAT activity markedly. Specifically, 5 μM FZ increased CAT activity to 270.6% and reduced MDA content to 75.8% of that in oxidatively stressed cells. Similarly, 5 μM DOF raised CAT activity to 292.3% and MDA content dropped to 81.6% of oxidatively stressed cells. Additionally, 10 μM vitamin C boosted CAT activity to 327% and reduced MDA content to 70% in oxidatively stressed cells (Figure 4d,e).

### 2.8. Effects of Compound Treatments on Skin Morphology

**Figure 5:**
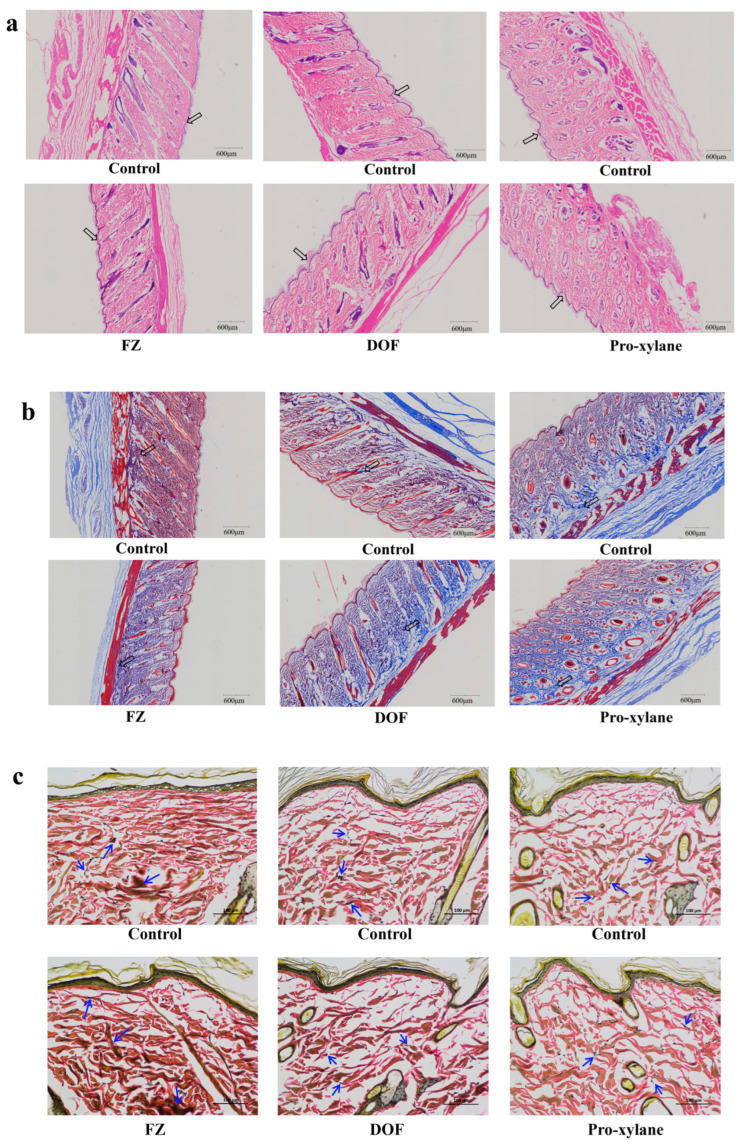
Effects of compounds on the morphology, collagen fibers, and elastic fibers of rat skin. (**a**) Hematoxylin and Eosin (H&E) stained images of rat skin. (**b**) Masson’s trichrome-stained images of rat skin. (**c**) Elastic Van Gieson (EVG) stained images of rat skin. Skin-stained pictures at a scale of 600 μm or 100 μm.

As the outermost layer of the skin, the epidermis serves to protect against external stimuli and prevent moisture loss. Hematoxylin-Eosin (HE) staining reveals fundamental morphological and structural characteristics of the skin. Rat skin treated with FZ or DOF showed no adverse reactions such as redness or inflammatory infiltration compared with the control group. Instead, the epidermis remained continuous and tightly arranged, suggesting that these compounds have a nourishing effect on the skin epidermis and can improve skin condition and barrier function, which decline with aging. In Figure 5a, the arrows point to the epidermal layer.

### 2.9. Effects of Compound Treatments on Skin Collagen Fibers

The dermal layer undergoes degenerative changes during aging, with the most prominent alterations occurring in the content and structure of collagen fibers. Unlike the abundant, tightly arranged collagen fibers in youthful skin, collagen fibers in aged skin appear fragmented and sparsely distributed [37]. Masson’s staining reflects the distribution and content of collagen fibers in the dermal layer of the skin. After treatment with FZ or DOF, the content of collagen fibers increased compared with the control group, with fibers being denser and more regularly shaped. In Figure 5b, the arrows point to the representative collagen fibers stained blue. This suggests that these compounds can improve collagen loss and fiber disruption associated with skin aging.

### 2.10. Effects of Compound Treatments on Rat Skin Elastic Fibers

Elastic fibers play a critical role in maintaining skin elasticity and compliance. They are a fibrous component of the dermal extracellular matrix, enabling the skin to return to its original state after stretching or deformation [38]. Despite constituting only 0.2% of the dry weight of the dermal layer, their water-absorbing capacity reaches up to 1000 times their own volume, regulating the hydration and compressibility of the dermis. Elastica van Gieson (EVG) staining specifically dyes elastic fibers black, revealing their structure and content within tissues. Skin treated with fructosazine, deoxyfructosazine, and hyaluronic acid showed evenly distributed elastic fibers with improved resilience and a higher content relative to the control group. In Figure 5c, the arrows point to some representative elastic fibers stained black. Elastic fibers provide the structural basis for skin elasticity, and a primary cause of wrinkle formation is the degeneration of elastic fibers. These results suggest that these compounds may decrease the degradation of elastic fibers associated with skin aging, thereby achieving an anti-wrinkle effect.

## 3. Discussion

In 2004, L’Oreal applied for patent protection for Pro-Xylane, which promoted the production of polyanionic glycosaminoglycans [39]. A relevant literature also made a detailed report [18]. Referring to the literature, a concentration of 1 mmol of Pro-xylane was selected for testing and comparison with FZ and DOF. Vitamin C has excellent antioxidant activity. It has been clinically tested to have anti-aging activity for the skin [40,41] and is widely used in cosmetics. Therefore, in the antioxidant activity test of FZ and DOF, vitamin C was selected as a reference.

The insights gained from the current investigation provided a detailed understanding of how FZ and DOF exert their beneficial effects on skin health and aging. Our results demonstrated that FZ markedly enhanced cell vitality, with the effect being more pronounced at a concentration of 5 μM, as reflected by an increase in cell vitality to 138.1% (Figure 2a). This enhancement was critical in combating skin aging, given that a decline in cell proliferation is a hallmark of aging skin. The compounds’ ability to promote the secretion of Col I and HA, while concurrently inhibiting the expression of MMP-1, is particularly noteworthy (Figure 3a–c).

This dual action not only supported skin hydration and elasticity but also prevented collagen degradation. The superior performance of FZ and DOF over Pro-xylane in this respect was highlighted by their more pronounced effects on the secretion of Col I and HA at lower concentrations. The compounds’ effect on catalase (CAT) activity and malondialdehyde (MDA) content underscores their antioxidant properties. FZ and DOF were found to significantly increase CAT activity and reduce MDA content, with FZ at 5 μM increasing CAT activity by 412.2% and decreasing MDA content to 28.2% (Figure 3d,e). The antioxidant activity of FZ and DOF is stronger than that of vitamin C. These findings are consistent with the compounds’ ability to mitigate oxidative stress, a key factor in skin aging.

The protective effects of FZ and DOF against oxidative damage were further evaluated in oxidatively stressed cells. Both compounds effectively scavenged reactive oxygen species (ROS) and reduced MMP-1 secretion, with FZ showing superior ROS scavenging ability compared with DOF (Figure 4b,c). These results suggest that FZ and DOF have the potential to reduce collagen and elastin breakdown, leading to improved skin firmness and wrinkle reduction. The compounds’ ability to lower the expression of SA-β-gal and maintain cell morphology in oxidatively stressed cells (Figure 4f,g) was also significant. This indicates that FZ and DOF may alleviate oxidative damage and delay cellular senescence, contributing to the overall health and appearance of the skin. In vivo studies revealed that FZ and DOF nourished the epidermis and enhanced the structure and morphology of collagen and elastic fibers in the dermis (Figure 5a–c). These effects were likely attributable to the compounds’ ability to stimulate the secretion of Col I and HA, while reducing MMP-1 expression, as observed in vitro.

In summary, this study demonstrated that FZ and DOF possess potent anti-aging properties, including the stimulation of cell vitality, promotion of collagen and hyaluronic acid secretion, inhibition of MMP-1 expression, and reduction of oxidative stress. These two compounds showed promise as effective active ingredients for the development of anti-aging pharmaceuticals and cosmetics, with the potential to improve skin health and appearance. Further research is warranted to elucidate the specific molecular mechanisms underlying these effects and to explore their long-term safety and efficacy in larger, more diverse populations.

## 4. Materials and Methods

### 4.1. Cell Culture and Oxidative Stress Model

NHDF cells (Shanghai Qingqi Biotechnology Co., Ltd., Shanghai, China) were cultured in DMEM medium (Hyclone, Logan, UT, USA) supplemented with 10% fetal bovine serum (Gibco, Brooklyn, NY, USA), 1% penicillin-streptomycin, and amphotericin B triple antibiotic solution (BI, Kibbutz Beit Haemek, Israel). Cells were maintained in a 37 °C incubator with 5% CO_2_. Oxidative stress was induced by incubating NHDF cells in a medium containing 5 µM H_2_O_2_ (Tianjin Damao Chemical Reagent Co., Ltd., Tianjin, China) for 24 h.

### 4.2. Drug Treatment of Normal Cultured Cells and Oxidatively Damaged Cells

NHDF cells were seeded at an appropriate density in cell culture flasks or dishes [42,43,44,45]. After 24 h to allow for cell attachment, the old medium was discarded. Fresh complete medium containing varying concentrations of the drugs was added for an additional 48 h of incubation. Following this treatment period, senescence markers were assessed.

In the case of stress-induced oxidative cellular damage, a drug pretreatment approach was adopted. Specifically, cells were first exposed to the drugs for 48 h, followed by the addition of fresh medium containing a final concentration of 5 µM H_2_O_2_ for an additional 24 h to establish an oxidative damage model. Upon completion of this treatment regimen, senescence indicators were measured in the oxidatively stressed cells.

### 4.3. Cell Viability Assay

NHDF cells were seeded in 96-well plates at a density of 3 × 10^3^ cells per well. After 24 h, the old medium was discarded and fresh complete medium containing different concentrations of the drugs was added for further culture for 48 h. After the drug treatment, the medium was removed, and 100 μL of serum-free medium containing 10% CCK-8 (Suzhou XinSaiMei Biotechnology Co., Ltd., Suzhou, China) was added to each well. After incubation for 2 h, the absorbance values at 450 nm were measured to calculate the cell viability.

### 4.4. Measurement of Col I, HA, and MMP-1 Secretion

NHDF cells were seeded in 96-well plates at a density of 3 × 10^3^ cells per well. After 24 h, the old medium was discarded, and fresh complete medium containing different concentrations of the drugs was added for further culture for 48 h. At the end of the treatment, the cell culture supernatant was collected. Subsequently, it was used to test the levels of Col I (Collagen Type I), HA (Hyaluronic Acid), and MMP-1 (Matrix Metalloproteinase-1) with the corresponding ELISA kits (name of the manufacturer, city, country). The secretion levels of Col I, HA, and MMP-1 were calculated according to the manufacturer’s instructions (Wuhan HuaMei Bioengineering Co., Ltd., Wuhan, China).

### 4.5. Measurement of Intracellular ROS Levels

The level of reactive oxygen species (ROS) was measured using the DCFH-DA method (Beijing Solarbio Life Science Co., Ltd., Beijing, China). NHDF cells were seeded in 96-well plates at a density of 3 × 10^3^ cells per well. After 24 h, the old medium was discarded and fresh complete medium containing different drug concentrations was added for a further 48 h of incubation. After treatment, intracellular ROS levels were tested and calculated according to the manufacturer’s instructions.

### 4.6. Measurement of CAT Activity and MDA Content

NHDF cells were seeded in cell culture dishes at a density of 5 × 10^5^ cells per dish. After 24 h, the old medium was discarded, and drug-containing medium was added for further culture for 48 h. At the end of the treatment, the cells were harvested. The cells per dish were added to 1 mL of extraction liquid and then sonicated in an ice bath. After that, the supernatant was collected following centrifugation at around 12,000 rpm at 4 °C for 10 min. The intracellular CAT activity and MDA levels were tested and calculated according to the manufacturer’s instructions (Suzhou Grice Biotechnology Co., Ltd., Suzhou, China).

### 4.7. Measurement of β-Galactosidase Expression

NHDF cells were seeded in 48-well plates at a density of 3 × 10^3^ cells per well. After 24 h of attachment, the old medium was discarded, and drug-containing medium was added for a further 48 h of incubation. After treatment, the β-galactosidase-positive senescent cells were stained and calculated according to the manufacturer’s instructions (Shanghai Beyotime Biotechnology Co., Ltd., Shanghai, China).

### 4.8. Animal Grouping and Drug Administration

All animal experimentation procedures adhered to the guidelines set by Dalian University of Technology’s Bioethics and Medical Ethics Committee. We used six male Sprague-Dawley (SD) rats (8-week-old, 200 ± 20 g) purchased from Liaoning Changsheng Biotechnology Co., Ltd. (Benxi, China). The animals were housed under controlled conditions (22 ± 3 °C, 12/12-h light/dark cycle) and randomly assigned to experimental groups. Two skin areas, each measuring 2 cm × 2 cm, were selected from the back of each rat, and the hair at the sampling sites was shaved. For each rat, one skin area was designated as the negative control, while the other was used as the drug-treated group. The skin in the drug-treated group was topically applied with 100 μL 0.25 mg/mL FZ, 0.24 mg/mL DOF, or 1.5 mg/mL Pro-xylane aqueous solution (containing 10% propylene glycol) daily for 3 weeks.

### 4.9. Sample Collection and Processing

After treatment, the required skin samples were excised, fixed with tissue fixative, and sent to Shanghai Beyotime Biotechnology Co., Ltd. for HE, Masson’s, and EVG staining.

### 4.10. Data Analysis

The data collected was analysed using GraphPad Prism software (8.4.0) to determine statistical significance.

## Data Availability

The data presented in this study are available on request from the corresponding author.

## Abbreviations

The following abbreviations are used in this manuscript:

FZ	Fructosazine
DOF	Deoxyfructosazine
NHDF	Normal human dermal fibroblast
mtDNA	mitochondrial DNA
PHAPs	Polyhydroxyalkylpyrazines
ROS	Reactive oxygen species
HA	Hyaluronic acid
Col	Collagens
MMP	Matrix metalloproteinase
CAT	Catalase
MDA	Malondialdehyde
SA-β-Gal	Senescence-associated β-galactosidase
HE	Hematoxylin-Eosin
